# Prevalence of Protozoa Species in Drinking and Environmental Water Sources in Sudan

**DOI:** 10.1155/2015/345619

**Published:** 2015-02-18

**Authors:** Salah Shanan, Hadi Abd, Magdi Bayoumi, Amir Saeed, Gunnar Sandström

**Affiliations:** ^1^Division of Clinical Microbiology, Department of Laboratory Medicine, Karolinska Institute, Karolinska University Hospital, Huddinge, 141 86 Stockholm, Sweden; ^2^Faculty of Medical Laboratory Sciences, University of Medical Sciences and Technology, P.O. Box 12810, Khartoum, Sudan

## Abstract

Protozoa are eukaryotic cells distributed worldwide in nature and are receiving increasing attention as reservoirs and potential vectors for the transmission of pathogenic bacteria. In the environment, on the other hand, many genera of the protozoa are human and animal pathogens. Only limited information is available on these organisms in developing countries and so far no information on their presence is available from Sudan. It is necessary to establish a molecular identification of species of the protozoa from drinking and environmental water. 600 water samples were collected from five states (Gadarif, Khartoum, Kordofan, Juba, and Wad Madani) in Sudan and analysed by polymerase chain reaction (PCR) and sequencing. 57 out of 600 water samples were PCR positive for protozoa. 38 out of the 57 positive samples were identified by sequencing to contain 66 protozoa species including 19 (28.8%) amoebae, 17 (25.7%) Apicomplexa, 25 (37.9%) ciliates, and 5 (7.6%) flagellates. This study utilized molecular methods identified species belonging to all phyla of protozoa and presented a fast and accurate molecular detection and identification of pathogenic as well as free-living protozoa in water uncovering hazards facing public health.

## 1. Introduction

Protozoa are eukaryotic cells distributed worldwide in nature and are receiving increasing attention as human and animal pathogens and potential vehicles for the transmission of bacteria in the environment. Unfortunately, one of two persons in the world is affected by waterborne or foodborne parasites [1]. The protozoa are among the most common parasitic pathogens present in environmental samples. They have multistage life cycles, consisting of an active trophozoite stage and a resistant stage (oocyst or cyst) excreted in feces that is capable of infecting new hosts [2].

The protozoa are subdivided into four phyla depending on their methods of locomotion. Mastigophora (flagellates) move by using one or more flagella. Sarcodina (amoebae) have extensions of the cytoplasm called pseudopodia assisting phagocytosis and motion in the organisms. Ciliophora (ciliates) move by means of cilia. Sporozoa (Apicomplexa) have no locomotion [3].

Over the past few decades, pathogenic enteric protozoa have been increasingly implicated in compromising the health of millions of people, mostly in developing countries. These protozoa contribute significantly to the staggering caseload of diarrheal disease morbidity encountered in these regions and are also significant concerns in industrialized countries despite improved sanitation [4].

*Acanthamoeba* is a genus of free-living amoebae (FLA), which are environmental eukaryotic cells distributed worldwide in nature [5, 6]. Life cycle of* Acanthamoeba* includes two stages, a feeding trophozoite and a dormant cyst stage.* Acanthamoeba* supports bacterial growth and survival and saves the bacteria from chlorination [7–9], increasing the risk of human illness caused by bacteria or* Acanthamoeba*.* Acanthamoeba* species are showing an increased role as human pathogens causing encephalitis, keratitis, pneumonitis, and dermatitis [10, 11] over the globe and the infection routes are mostly from the environment.

*Cryptosporidium* species and* Giardia intestinalis* are major pathogens in the waterborne transmission of infections and they are able to persist in the environment due to the robustness of the oocysts and cysts. The occurrence of these microorganisms in different types of water has been confirmed, and a considerable number of waterborne outbreaks have been reported worldwide [12–14].

In Sudan,* Cryptosporidium* species is an important cause of diarrhea in children, and it is suggested that intrafamilial spread occurs [15]. The highest prevalence of diarrheal diseases was recorded among Port Sudan children (15.5%), followed by children living in areas where people draw water from unrectified hafirs (13.5%) and finally children living in areas where water is drawn from rectified hafirs (6.0%) [16]. However, there is no information about the presence of the protozoal species except* Cryptosporidium*. The aims of this study were to detect protozoa from water samples by polymerase chain reaction (PCR) using primer (P-SSU-342f and Medlin B) and identify species of the protozoa by sequencing.

## 2. Material and Methods

### 2.1. Sample Collection

**Figure 1:**
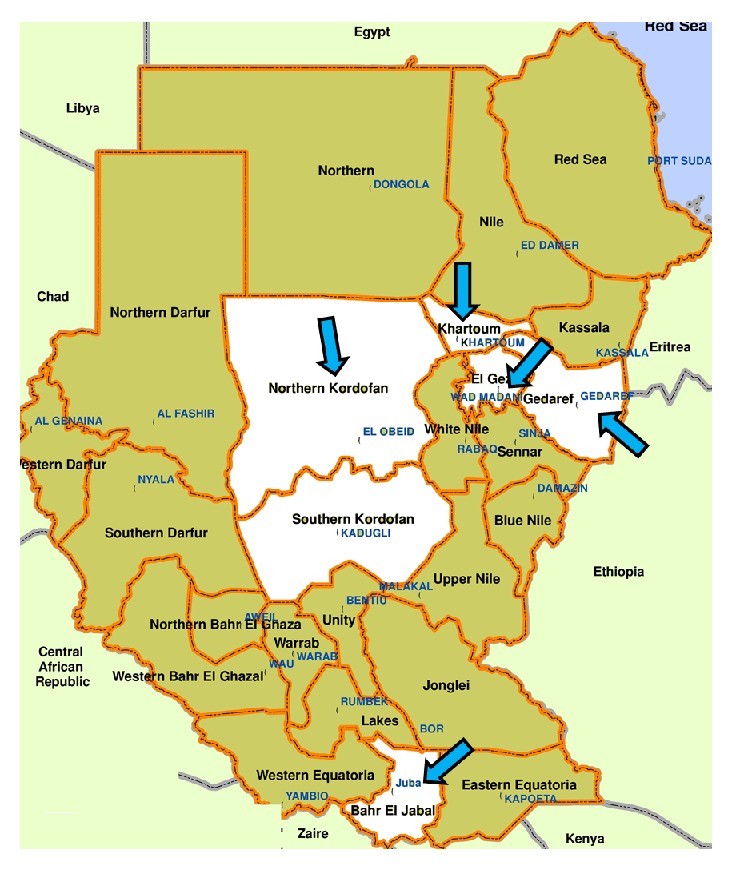
White color with arrows showing the surveyed areas in Sudan and South Sudan.

600 samples were collected from water sources (zeer, hafir, tank, lake, and stream) that belonged to different areas (Gadarif, Khartoum, Kordofan, Juba, and Wad Madani) in Sudan for a one-year period in 2008 (Table 1, Figure 1).

The hafir (an underground water reservoir designed for storing rain water carried by streams and used for domestic water supply and for agricultural purposes in rural areas in the Sudan) [17] is one of the most common sources of water in peripheral areas, and different types of materials (contaminants) accumulate in it (due to erosion), as well as the feces from wild and domestic animals. The zeer is a traditional Sudanese storage jug made of baked clay, commonly used to keep drinking water. Water samples were collected into sterile 50 mL tubes and transported to the laboratory at Microbiology Department, University of Medical Sciences and Technology, Khartoum.

### 2.2. DNA Extraction

The samples were centrifuged for 10 min at 4000 rpm. The supernatant was removed and the sediment was used for DNA extraction. The DNA was extracted using routine proteinase K procedures using Qiagen DNA mini kit (Qiagen, Valencia, CA, USA).

### 2.3. DNA Amplification

A protozoa-specific forward primer (P-SSU-342f) and Eukarya-specific reverse primer (Medlin B) targeting 18S rRNA gene were used. The forward primer was P-SSU-342f (5′ CTTTCGATGGTAGTGTATTGGACTAC-3′) and the reverse primer was Medlin B (5′-TGATCCTTCTGCAGGTTCACCTAC-3′), [18, 19] in PCR to obtain a product of about 1,360 bp. The PCR reaction performed at 95°C for 10 min, 35 cycles at 94°C for 1 min 50°C for 1 min 72°C for 1 min, final extension at 72°C for 10 min using 0.4 *μ*M of each primer. The PCR amplification step was carried out and the final PCR reaction mixture was divided into two parts. One part was used for 2% agarose gel electrophoresis with ethidium bromide staining and the other part was used for DNA sequencing.

*A. castellanii* was used as a positive control. Detection sensitivity was performed by serial dilution of* A. castellanii* (10 cells/mL to 10^6^ cells/mL).

### 2.4. Sequencing

After samples collection, DNA extraction, and PCR analysis, direct sequencing of the protozoa 18S rRNA gene was performed using purified nested PCR products. The sequencing was carried out by MWG operon (MWG operon, Germany). The nucleotide sequences were then edited and aligned using Blast program; the sequencing confirmed protozoal species according to the GenBank accession numbers for their nucleotide sequences.

### 2.5. Statistical Analysis

*χ* ^2^ test was used to verify differences in the existence of protozoa in water samples collected from different sources. A *P* value of ≤0.05 was considered statistically significant.

## 3. Results

**Figure 2:**
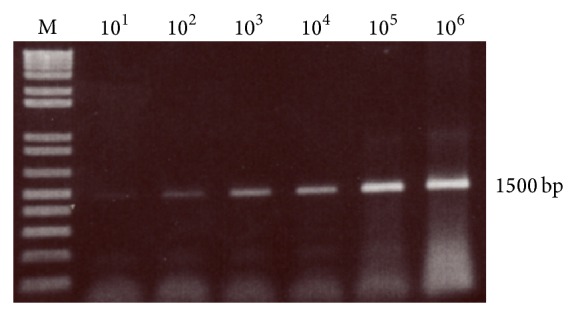
Detection sensitivity test of PCR of amoeba 18S rDNA.

**Figure 3:**
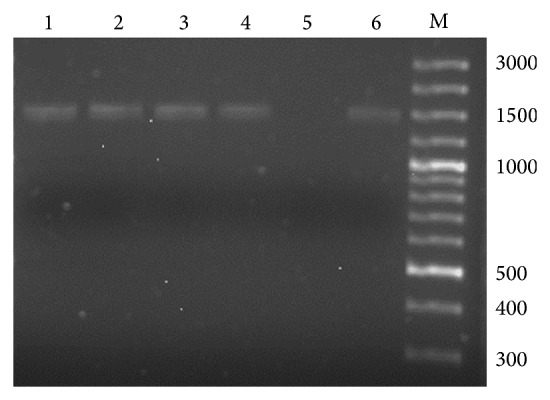
Representative agarose gel (2%) electrophoresis of PCR products of protozoal 18S rRNA. Lanes 1 to 4 samples contain protozoa; 5 and 6 are negative and positive controls (approximately 1,360 bp). M is molecular mass markers (1 bp).

The current study collected environmental water samples and extracted, amplified, visualised, and sequenced the PCR products using Blast program. A detection limit of 10 cells was found for* Acanthamoeba* according to the serial dilutions (Figure 2). The PCR amplification of the genomic DNA extracted from water samples produced a single band of the expected size of 1360 bp (Figure 3). Out of 600 water samples, 57 positive samples were detected by PCR and gel electrophoresis utilizing primer targeting 18S rRNA gene of the protozoa (Table 1).

The PCR positive samples were sequenced, 38 samples passed sequencing, and 19 samples failed to pass the sequencing. The sequenced samples yield 66 species of protozoa which belonged to four phyla, which were amoebae 28.8%, Apicomplexa 25.7%, ciliates 37.9%, and flagellates 7.6%.

The identified amoebae were* Acanthamoeba castellanii, Capsaspora owczarzaki, Dictyostelium purpureum, Entamoeba dispar*, and* Naegleria gruberi*. The Apicomplexa were* Ascogregarina taiwanensis, Blastocystis hominis, Cryptosporidium muris, Cryptosporidium parvum, Neospora caninum, Theileria parva*, and* Toxoplasma gondii*. The ciliates were* Ichthyophthirius multifiliis, Oxytricha trifallax*, and* Stylonychia lemnae*. The flagellates were* Perkinsus marinus* and* Trichomonas vaginalis* (Table 2).

## 4. Discussion

Unsafe water and poor sanitation and hygiene have been reported to rank third among the 20 leading risk factors for health burden in developing countries, including Sudan [20].

Hafir, tank, zeer, lake, and stream are the main sources of drinking water in Sudan, especially in the rural areas, and not only used for humans. Consumption is also used for animals and accordingly this is the common source of contamination with protozoa and other harmful microorganisms. Although there is much scientific literature available concerning the association of protozoa with waterborne diseases, this is not the case in Sudan.

Our study has pioneered the use of a PCR-based molecular test to search for potentially disease causing protozoa in drinking water, resulting in the detection of four phyla of well-documented pathogenic protozoa. This is the first study in Sudan investigating the detection and identification of protozoa found in drinking water. The result will provide vital information regarding the protozoa found at different geographic locations, thus facilitating correlation of the identified organisms with the clinical phenotypes of infectious disease prevalent among the population inhibiting specific geographical locations.

The result in this study demonstrated that protozoa are common in all water sources, especially in Gadarif, Kordofan, and Juba, and distributed even in all drinking water sources: hafirs, zeers, lakes, tanks, and streams. 66 species of protozoa were identified by sequencing and they include amoebae species (28.8%), Apicomplexa species (25.7%), ciliates (37.9%), and flagellates (7.6%).

In this context, studies by Leiva et al. [21] and Tsvetkova et al. [22] reported that the amoebae were found in 43% and 61%, respectively, of the water samples. Moreover, in a recent study in Turkey, only three species of free-living amoeba,* A. castellanii, A. polyphaga*, and* Hartmannella vermiformis*, were identified from tap water [23]. In addition,* Cryptosporidium, Giardia*, and* Acanthamoeba* were isolated from stations of recreational lakes in Malaysia [24].

However, these studies detected a limited number of amoebae,* Cryptosporidium*, and* Giardia* only in water samples compared to our study that identified 49 microorganisms comprising 16 species belonging to four different phyla of protozoa, which gives an alert about the risk of water contamination since most of these protozoa are human pathogens or zoonotic parasites.

To our knowledge, no proper studies were performed in Sudan searching for protozoa in water sources. However, Adam et al. [15] reported that* Cryptosporidium* was an important cause of diarrhea in children in Sudan. Most of the international studies in the field detected limited numbers of protozoal species such as* Cryptosporidium* and* Giardia* [25, 26].

It is well known that a complex interaction between organisms is found in aquatic environment, especially amoebae and bacteria. Free-living amoebae are eukaryotic cells found in nature and including several genera such as* Acanthamoeba, Balamuthia, Naegleria*, and* Sappinia*. It has been shown that* Acanthamoebae* benefits from* E. coli* and* Klebsiella aerogenes* as food. In contrast, the role of* Acanthamoebae* as hosts and vectors for bacteria has been proposed for many pathogenic bacteria [27].

The interaction between bacteria and amoebae is very complex. Output of that interaction is depending on whether the interacted bacterium is extracellular or intracellular and if it possesses a type three secretion system (TTSS) or not since TTSS effector proteins are observed to affect strongly output of the interaction [27]. Extracellular bacteria cannot multiply inside amoeba cell and TTSS possessing extracellular bacteria such as* P. aeruginosa* kill the amoebae. However, the extracellular bacterium* E. coli* does not possess TTSS and therefore it is ingested as food by the amoebae.

An intracellular bacterium multiplies inside amoeba cell. The intracellular bacterium that does not possess TTSS* F. tularensis* multiplies symbiotically inside the amoebae, while TTSS possessing intracellular bacterium* E. coli* K1 and* Shigella flexneri* lyse the amoebae according to activation of TTSS [27].

Facultative intracellular bacteria such as* Vibrio cholerae* are able to multiply in water and inside the environmental phagocytic eukaryotes such as free-living amoebae. Therefore, presence of the amoebae in water will enhance presence of bacteria such as* V. cholerae* [7, 28, 29]. Surprisingly, in this context,* Acanthamoeba* species and* V. cholerae* were detected from the same water samples collected from different water sources in Sudan [27, 30] and Newsome et al. isolated an amoeba naturally harboring a distinctive* Legionella* species [31, 32].* V. cholerae* and* Legionella pneumophila* are well known as causative agents for the waterborne diseases cholera and legionellosis.

The protozoa have a double infectious role in public health. They can cause infections by themselves such as amoebiasis, cryptosporidiosis, giardiasis, amoebic encephalitis, and amoebic keratitis and they can act as a vector to their intracellular bacteria to cause multiple infections of protozoa and bacteria [31].

In addition to our findings about the identified protozoa in this paper, Fletcher et al. [33] reviewed that* G. intestinalis, Cryptosporidium* spp., and* Entamoeba* spp. were the most commonly reported protozoa associated with enteric infections and were associated mainly with food- and waterborne outbreaks. These enteric protozoa are isolated frequently from diarrheal patients in developing regions such as Asia and Sub-Saharan Africa [33]. However, recently Muchesa et al. detected 12.8%* Acanthamoeba* from 172 wastewater samples [34]. Our study presents the first report about detection of the following protozoa:* A. castellanii, T. gondii, C. owczarzaki, B. hominis, C. muris*, and* T. vaginitis* from zeers found in Gadarif, Juba, and Khartoum.

Overall prevalence of detected protozoa by PCR was 9.5% and the prevalence in Gadarif, Juba, Khartoum, Kordofan, and Wad Madani was 14.3%, 14.0%, 9.1%, 14.0%, and 2.8%, respectively. The prevalence of identified protozoa in Gadarif, Juba, Khartoum, and Kordofan was not statistically significant by *χ* ^2^ test (*P* > 0.05). Moreover, the prevalence of identified protozoa in Wad Madani compared to those of Gadarif, Juba, and Kordofan was highly statistically significant (*P* values were 0.0012, 0.0007, and 0.0003, resp.). But the prevalence of identified protozoa in Wad Madani and Khartoum was not statistically significant; *P* value was 0.1210.

This result uncovers the danger coming from water to spread infections between populations and our study presented a fast and accurate molecular detection and diagnosis of protozoa species in water.

## Figures and Tables

**Table 1:** Number of positive samples by PCR utilizing primer targeting protozoal 18S rRNA gene.

Location	Positive samples	Negative samples	Total	Prevalence
Gadarif	19	114	133	14,3%
Juba	12	74	86	14,0%
Khartoum	6	60	66	9,1%
Kordofan	14	86	100	14,0%
Wad Madani	6	209	215	2,8%

Total	57	543	600	9,5%

**Table 2:** Identified protozoa species by sequencing found in water samples collected from different sources and locations.

Location	Source	Number of identified protozoa							
		Amoebae	Number	Apicomplexa	Number	Ciliates	Number	Flagellates	Number
Gadarif	Zeer	*A. castellanii *	2	*T. gondii *	1		0		0
	Hafir	*C. owczarzaki *	1	*C. muris * *N. caninum *	1 1	*I. multifiliis *	1	*P. marinus *	2
		*D. purpureum *	1			*O. trifallax *	4		
		*A. castellanii *	2			*S. lemnae *	3		
	Tank	*A. castellanii *	1	*C. muris *	2	*S. lemnae *	2		0
		*E. dispar *	1	*T. parva *	1	*O. trifallax *	2		

Juba	Lake	*A. castellanii *	3	*C. muris *	2	*S. lemnae * *O. trifallax *	1 3		0
				*N. caninum *	1				
				*A. taiwanensis *	1				
	Zeer	*C. owczarzaki *	1	*B. hominis *	1	*O. trifallax *	1		0
		*A. castellanii *	1						

Khartoum	Lake	*A. castellanii *	1	*T. gondii *	1	*O. trifallax *	2		0
	Zeer	0	0	*C. muris *	1	0	0	*T. vaginitis *	1

Kordofan	Hafir	*D. purpureum *	1	*T. gondii * *A. taiwanensis *	2 1	*O. trifallax * *S. lemnae *	4 2	*P. marinus *	2
		*N. gruberi *	1						
		*E. dispar *	1						

Wad Madani	Stream	*A. castellanii *	1	*C. parvum *	1	0	0		0
		*C. owczarzaki *	1						

Total			19		17		25		5
